# Visual outcomes and prognostic factors in open-globe injuries

**DOI:** 10.1186/s12886-018-0804-4

**Published:** 2018-06-08

**Authors:** Azusa Fujikawa, Yasser Helmy Mohamed, Hirofumi Kinoshita, Makiko Matsumoto, Masafumi Uematsu, Eiko Tsuiki, Kiyoshi Suzuma, Takashi Kitaoka

**Affiliations:** 1 Department of Ophthalmology and Visual Sciences, Graduate School of Biomedical Sciences, Nagasaki University, 1-7-1 Sakamoto, Nagasaki, Nagasaki 852-8501 Japan; 2 Department of Ophthalmology, EL-Minia University Hospital, EL-Minia, Egypt; 3 Department of Ophthalmology and Visual Sciences, Graduate School of Medicine Kyoto University, Kyoto, Japan

**Keywords:** Open-globe injury, Vitrectomy, Retinal detachment, Penetrating keratoplasty

## Abstract

**Background:**

Ocular trauma is an important cause of visual loss worldwide. Improvements in our knowledge of the pathophysiology and management of ocular trauma during the past 30 years, in conjunction with advances in the instrumentation and techniques of ocular surgery, have improved the efficacy of vitreoretinal surgery in injured eyes. The aim of the current study was to determine the visual outcomes and prognostic factors of open-globe injuries in the Japanese population.

**Methods:**

Retrospective study of 59 eyes of 59 patients presented with open globe injuries between September 2008 and March 2014 at Nagasaki University Hospital was conducted.

Demographic factors including age, gender, and clinical data such as cause of injury, presenting visual acuity (VA), location of injury, type of injury, lens status, presence of intraocular foreign body, types of required surgeries, and final VA were recorded. According to the classification of Ocular Trauma Classification Group, wound location was classified into three zones. Chi-square test was used to compare presented data.

**Results:**

Out of the 59 patients, 46 were placed in the Light Perception (LP) group, and 13 were placed in the No Light Perception (NLP) group. Work-related trauma was the most common cause (27 eyes) followed by falls (19eyes). Work-related trauma was common in males (*P* = 0.004), while falls was significantly common in females (*P* = 0.00001). Zone III injuries had statistically significantly poor prognostic factor compared to other zones (*P* = 0.04). All cases of NLP group (100%) presented with rupture globe. Poor VA at first visit (*P* = 0.00001), rupture globe (*P* = 0.026), history of penetrating keratoplasty (PK) (*P* = 0.017), retinal detachment (RD) (*P* = 0.0001), vitreous hemorrhage (VH) (*P* = 0.044), and dislocation of crystalline lens (*P* = 0.0003) were considered as poor prognostic factors.

**Conclusion:**

Poor VA at first visit, rupture globe, zone III injuries, history of penetrating keratoplasty, RD, VH, and dislocation of crystalline lens were found to be poor prognostic factors. PPV had a good prognostic value in open globe injuries associated with posterior segment involvement.

## Background

Ocular trauma is a prominent cause of visual disability and, depending on the sample population, can contribute up to 65% of the cases of unilateral blindness worldwide. The burden of blindness is related to both its inevitable effect on the quality of life and the loss of productivity that subsequently occurs in these subjects [1, 2]. Mechanical trauma to the eye has been classified by the Birmingham Eye Trauma Terminology (BETT) and Ocular Trauma Classification Group and subdivided it into open and closed globe injuries [3].

An open-globe injury is defined as a full thickness wound of the eye wall (full injury of the sclera, cornea, or both) with this vision-threatening condition often leading to blindness. Although there has been considerable effort to prevent this type of blindness, it remains common around the world, with an annual global incidence rate of 3.5/100,000 persons [4]. There is worldwide interest in the epidemiology of ocular trauma [2]. Different studies have reported varying proportions of open versus closed globe injury [5–7]. Although public health campaigns have been organized to prevent eye injuries, unfortunately, open-globe injuries are still too frequent. Moreover, it has been shown that open-globe injuries result in more hospitalization and a poorer visual outcome compared to closed globe injuries [8, 9].

Improvements in our knowledge of the pathophysiology and management of ocular trauma during the past 30 years, in conjunction with advances in the instrumentation and ocular surgery techniques, have improved the efficacy of vitreoretinal surgery in injured eyes [10].

Achieving or maintaining useful vision is dependent upon several prognostic factors, such as the severity of the initial trauma, involvement of ocular structures, preoperative visual acuity, and both a timely diagnosis and treatment [10, 11].

The aim of the current study was to determine the visual outcomes and prognostic factors of open-globe injuries in the Japanese population.

## Methods

This retrospective study reviewed the records of all subjects who sustained an open-globe injury and were examined at Nagasaki University Hospital between September 2008 and March 2014.

Reviews of the patients’ medical charts included the initial ophthalmology consultation notes, hospital records, details of the primary and subsequent surgical interventions, and outpatient follow-up records. During the review of the records, demographics, including age and gender, wound characteristics (i.e., mechanism, causes, sizes, and locations), and visual acuity (VA) (presenting and final VA), were collected. The final VA was defined as the VA at the end of the follow-up. Associated ocular damage (i.e., vitreous hemorrhage (VH), retinal detachment (RD), intraocular foreign body (IOFB), lens status, and endophthalmitis) was also evaluated.

Based on the Birmingham Eye Trauma Terminology, the mechanisms of injury were classified as rupture, penetration, IOFB, perforation, and mixed injury [3]. In cases in which there was a high clinical suspicion of an IOFB that could not be confirmed by clinical examination or in which the media opacity prohibited any examination of intraocular structures, ancillary testing with X-rays, computed tomography, or echography were used to classify the injuries.

Patients were divided into groups according to the real size of the wound (in mm). The 4 classifications used included wounds that were smaller than 5 mm, 5–10 mm, 10–15 mm, and larger than 15 mm.

Distance VA was tested using a Landolt C acuity chart. If the VA improved when using a pinhole, this was recorded as the VA at the initial examination. Details of the primary and subsequent treatments and final VAs were also collected. The initial VA was divided into the following 6 categories: acuity 20/40 or better, between 20/40 to 20/400, between 20/400 to counting fingers (CF), hand movement (HM), light perception (LP), and no light perception (NLP). The outcome, which was defined as the VA measured at the last visit, was divided into 2 categories: ocular survival (with VA ranging between 20/20 and LP) (LP group) and NLP (NLP group). The visual acuity of NLP was confirmed using a bright light source, such as an indirect ophthalmoscope. This light source was set at the highest intensity during which time the fellow eye was completely occluded. A cross-sectional analysis was performed on all patient data in order to investigate the correlation between the initial and final VA. As part of this investigation, we used a value of 1/400 VA (logMAR = 2.6) to represent the vision of the CF patients, with the extrapolated values of 2.7, 2.8, and 2.9 logMAR used to represent HM, LP, and NLP, respectively.

Wound locations were classified according to the Ocular Trauma Classification Group [3]. Zone I injuries were confined to the cornea and limbus, zone II injuries involved the anterior 5 mm from the limbus (not extending into the retina), and zone III injuries extended to the posterior by more than 5 mm from the limbus. In cases of multiple corneoscleral openings, the zone was defined according to the most posterior opening. In cases of IOFBs, the zone was defined at the specific entry site. For perforating injuries, however, the zone was defined by the most posterior defect, which was generally the exit site. While all of the zones of the injury were determined at the time of the initial examination, in some cases, the exact extent of the injury was more accurately determined during the surgical intervention, which led to further identification and revision of the zone of injury.

After collecting all of the records, the data were evaluated for the influences of the initial VA, wound location and size, mechanism of injury, and associated ocular tissue damage on the visual survival rates. This study was approved by the Institutional Review Board (IRB) of the Nagasaki University Hospital and adhered to the tenets of the Declaration of Helsinki.

Statistical Methods:

A Student’s two-tailed t-test was used to compare the quantitative variables, while the chi-square test was used to compare the categorical data. Values of *p* < 0.05 were considered statistically significant.

## Results

### Patient demographics

**Fig. 1:**
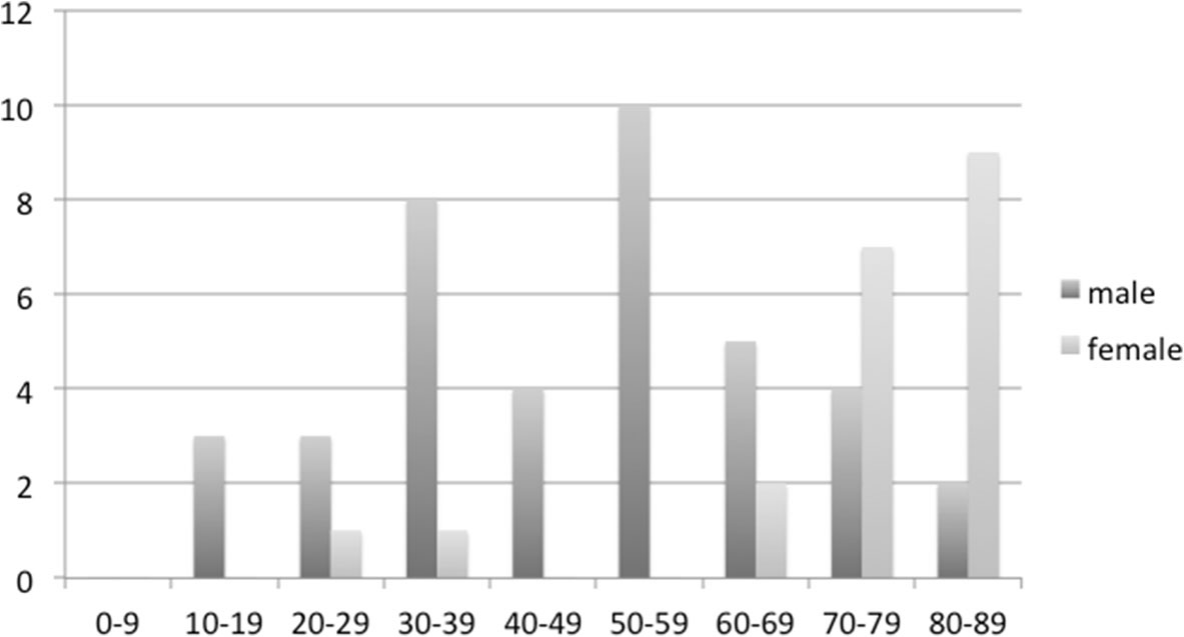
Sex and age distribution of the patients with open-globe injury. The patient group consisted of 39 (66.1%) males and 20 (33.9%) females, with a male:female ratio of nearly 2:1

This study evaluated 59 patients (46 in the LP group and 13 in the NLP group). The mean age was 56.7 ± 21.8 years in the LP group and 62.3 ± 21.7 years in the NLP group, with no significant difference found between the two groups (*p* = 0.21). Figure 1 presents the details for the age and gender distribution. Only 1 case was younger than the age of 16 years. The patient group included 39 (66.1%) males and 20 (33.9%) females, resulting in a ratio of nearly 2:1.

None of the patients presented with a bilateral open-globe injury. The injuries occurred in 27 right eyes and in 32 left eyes, with no significant difference with regard to the side.

### Cause of injury

**Fig. 2:**
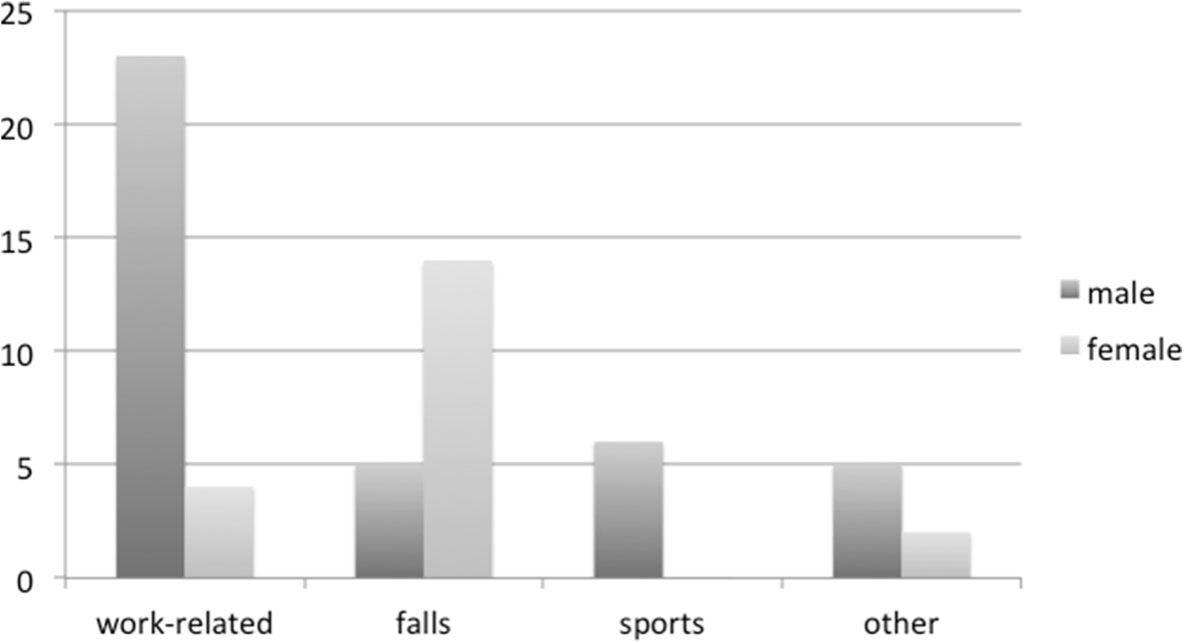
Cause of injury: Work-related trauma was more common in males (*p* = 0.004), while falls were more common in females (*p* = 0.00001)

Work-related trauma (27 [45.8%] eyes) was the most common cause of the injury, which was followed by falls (19 [32.2%] eyes). Work-related trauma was common in males (*p* = 0.004), while falls were common in females (*p* = 0.00001) (Fig. 2). The mean ages were 51.3 ± 18.1 years for the work-related trauma and 61.1 ± 8.8 years for the falls. There were 6 (10.2%) eye injuries related to sports injuries, and 2 (3.4%) eye injuries caused by car accidents.

### Mechanism of injury

All 13 (100%) eyes in the NLP group and 28 (60.8%) eyes in the LP group presented with a ruptured globe. Ruptured globe was a statistically significant poor prognostic factor (*p* = 0.026) (Table 1).

**Table 1:** Final visual outcomes and prognostic factors

Final VA	LP	NLP	Total	*P* value
n	46	13	59	
Age	56.7	62.3		0.21
Sex				
Male	30	9	39	0.67
Female	16	4	20	
Trauma eye				
Right	23	4	27	0.22
Left	23	9	32	
Type of injury				
Penetrating	6	0	6	0.026
Rupture	28	13	41	
Foreign body	12	0	12	
Location				
Zone I	21	4	25	0.34
Zone II	21	5	26	0.64
Zone III	4	4	8	0.04
Initial Visual Acuity				
20/40 ≤	5	0	5	0.0001
20/400 < 20/40	3	0	3	
CF < 20/400	5	0	5	
HM	12	0	12	
LP	20	5	25	
NLP	1	8	9	
Size of injury				
≤ 5 mm	17	0	17	0.0078
5–10 mm	21	6	27	
10–15 mm	8	6	14	
15 < mm	0	1	1	
Status of the lens				
Aphakia	2	2	4	0.34
Phakia	31	7	38	
Pseudophakia	13	4	17	
Dislocation of the lens				
Yes	5	6	11	0.00025
No	26	1	27	
History of PKP				
Yes	3	4	7	0.017
No	43	9	52	
Retinal detachment				
Yes	10	10	20	0.0001
No	36	1	37	
Unknown	0	2	2	
Vitreous Hemorrhage				
Yes	27	10	37	0.0443
No	19	1	20	
Unknown	0	2	2	
Primary operation with PPV				
Yes	26	3	29	0.033
No	20	10	30	

All 13 (100%) eyes in the NLP group and 28 (60.8%) eyes in the LP group presented with a ruptured globe. Ruptured globe was a statistically significant poor prognostic factor (p = 0.026)

*VA* Visual acuity, *LP* Light perception, *NLP* No light perception, CF counting fingers, *HM* hand movement, *PK* Penetrating Keratoplasty, *RD* Retinal detachment, *VH* Vitreous hemorrhage, *PPV* Pars plana vitrectomy

Posterior segment IOFBs were observed in 12 (20.3%) eyes, with all of these patients belonging to the LP group (26.1%). A metallic IOFB was found in 11 cases, while 1 case had a concrete IOFB. In these patients, IOFB removal was the primary procedure performed, with 3 IOFBs located in the anterior chamber, 2 in the vitreous cavity, 6 in the peripheral retina, and 1 nasal relative to the optic disc in the posterior pole of the eye. IOFB removal was successful in all cases.

Penetrating trauma occurred in the remaining 6 eyes of the LP group (13.1%). However, neither penetrating trauma nor IOFB exhibited any significant predictive factors.

### Location of injury

Zone III injuries were more common in the eyes with a final VA of NLP (4 [30.8%] of 13 eyes) as compared to eyes with a final VA of LP or better (4 [8.7%] of 46 eyes). Zone III injuries were found to be a statistically significant poor prognostic factor for visual outcome (*p* = 0.04). Zone I injuries were found in 25 (42.4%) eyes (21 in the LP group and 4 in the NPL group), while zone II injuries occurred in 26 (44.1%) eyes (21 in the LP group and 5 in the NPL group). There were no statistically significant predictive factors for visual outcomes found for zones I and II.

### Size of the injury

Patients with a wound that was smaller than 5 mm had a statistically significant better prognosis than patients with wounds that were larger than 5 mm (*p* = 0.0078).

### Associated ocular damage

Crystalline lens expulsion, which occurred in 11 eyes (5 in the LP group and 6 in the NLP group), was a poor prognostic factor for visual outcome (*p* = 0.0003). However, results showed that phakia, aphakia, and pseudophakia were not significant predictive factors for visual outcome.

A history of penetrating keratoplasty (PK) was found in 4 (30.8%) of 13 eyes in the NLP group (30.8%) and in 3 (6.5%) eyes in the LP group. A past history of PK was a statistically significant poor prognostic factor for visual outcome (*p* = 0.017).

Both RD and VH were statistically significant poor prognostic factors for visual outcome (*p* = 0.0001 and *p* = 0.044, respectively). Pars plana vitrectomy (PPV) was performed in 11 RD cases. Retinal re-attachment with a good visual outcome was found in 8 cases (LP group), while 3 cases (NLP group) failed to obtain a good visual outcome primarily due to a severely torn retina. PPV was additionally performed as the primary procedure in 22 cases that presented with VH. Of these, 3 cases (NLP group) failed to gain a good visual outcome, while 19 cases (LP group) did achieve a good visual outcome after the procedure.

Our results also showed that there were no significant predictive factors or any statistically significant differences between the two groups in this study for other associated ocular damage, such as choroidal hemorrhage and hyphema. In addition, we did not find a single case of traumatic or postoperative endophthalmitis in this study.

### PPV

PPV was performed as the primary operation in 29 (49.2%) cases. The most common indications were VH (22 [75.9%] of 29 eyes) and RD (11 [37.9%] of 29 eyes), with 26 (56.5%) patients undergoing the procedure in the LP group and 3 (23.0%) patients in the NLP group. Eyes that underwent PPV were significantly more likely to achieve a final vision of LP or better as compared to those that did not (*p* = 0.033). Furthermore, in the eyes that achieved a final VA of LP or better, those that underwent PPV were found to have slightly better visual outcomes (0.81 logMAR) versus those that did not undergo the procedure (1.1 logMAR). In 2 cases where the globes were severely disorganized in addition to a fear of the subsequent occurrence of sympathetic ophthalmia, enucleation was performed as the primary procedure.

### Visual acuity

**Fig. 3:**
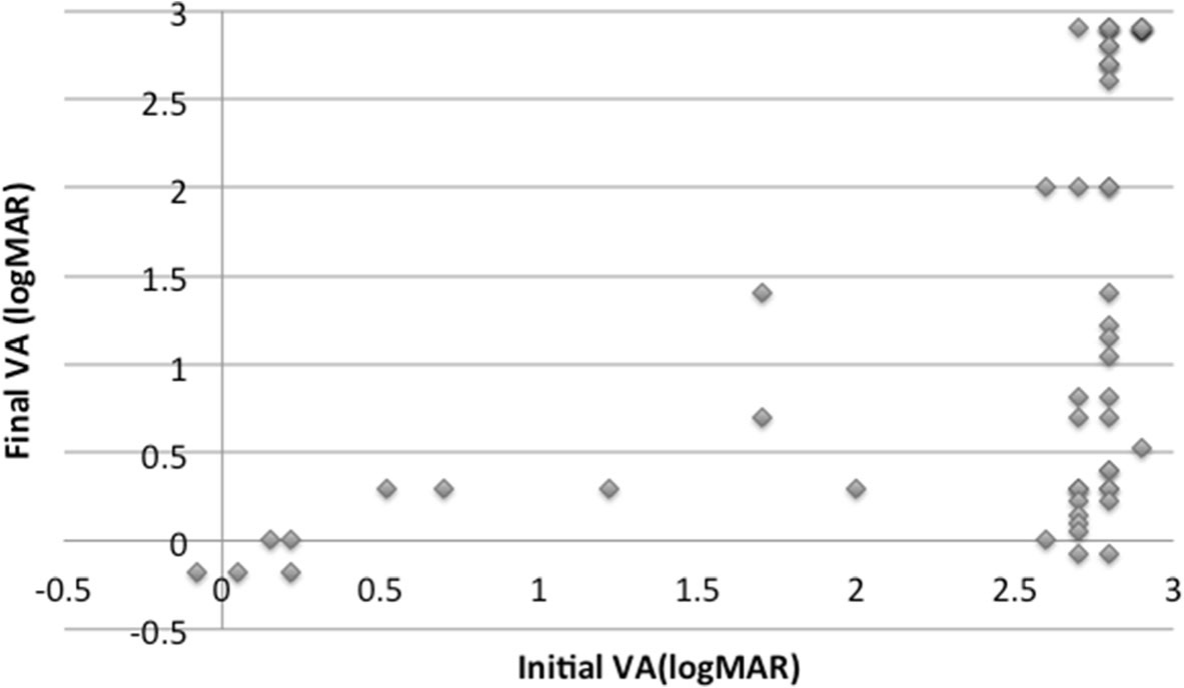
Correlation between the initial and final VA. Even when the initial VA was poor, there was variation in the final VA

Poor VA at the first visit (*p* = 0.00001), a ruptured globe (*p* = 0.026), history of PK (*p* = 0.017), zone III injuries (*p* = 0.04), RD (*p* = 0.0001), VH (*p* = 0.044), and expulsion of the crystalline lens (*p* = 0.0003) were all determined to be poor prognostic factors. Patients with a wound that was smaller than 5 mm had a significantly better VA than those groups that had wounds that were larger than 5 mm (*p* = 0.0078). Eyes that were first treated with PPV were significantly more likely to achieve a final vision of LP or better (*p* = 0.033). Figure 3 shows the correlation between the initial and final VA. As seen in this figure, even if the initial VA was poor, there was variation in the final VA.

## Discussion and conclusion

This study evaluated the visual outcomes after open-globe injuries in Japanese patients and tried to identify the possible risk factors and the prognostic factors responsible for the final visual outcomes. To the best of our knowledge, this is the first study that has examined the prognostic factors of open-globe injuries in the Japanese population.

The mean age of our study populations was 56.7 ± 21.8 years in the LP group and 62.3 ± 21.7 years in the NLP group. These values are higher than those that have been reported in other studies [11–14]. Additionally, the mean age of the work-related trauma group was 51.3 ± 18.1 years while that for the falls group was 61.1 ± 8.8 years. This variability may be due to inter-population differences in the culture, lifestyle, mean lifespan, occupation, and socioeconomic status. Unlike other studies that have reported finding a prevalence of children with eye traumas [11–14], we only encountered 1 case of a patient who was younger than 16 years of age.

The male predominance (male:female = 2:1) observed in our current study is in agreement with the results of several previous studies [12, 14–17]. As previously speculated, this most likely indicates that males probably have a higher risk of being exposed to dangerous situations in the workplace or during outdoor activities, as well during gender-based behavior [16].

Occupational injuries predominate in older age groups, with work-related trauma (27 [45.76%] eyes) followed by falls at home (19 [32.2%] eyes) the most common causes of trauma that result in open-globe injuries. In the relatively younger age groups, the trauma was related to sports injuries (6 [10.2%] eyes).

In line with our results, the previous literature has also reported that the most frequently associated cause of trauma was occupational injury [11, 13, 18, 19]. Other studies have confirmed that the majority of open-globe injuries occur in the home, ranging from 38 to 71% of the cases [20, 21]. However, Tok et al. [22] and El-Sebaity et al. [5] reported that injuries at home were less prevalent than those outside the home in pediatric cases. However, work-related trauma remains an important cause of avoidable and predominantly monocular visual morbidity, due to the fact that the majority of injuries are the result of not following proper safety precautions. Although the use of safety precautions had no effect on the final visual acuity, safety precautions are advised for all practical purposes as a means of preventing injuries, ocular or otherwise [18].

Although one of the important causes of open-globe is related to car accidents, our current study found that only 2 (3.4%) cases were due to an automobile accident. Thevi et al. confirmed that road accidents are the second cause of open-globe injuries (*n* = 17, 32.7%) [18].

The most frequent mechanism of injury was rupture injury (69.5%), which was followed by IOFBs (20.3%) and penetrating injury (10.2%). All 13 (100%) eyes in the NLP group presented with a ruptured globe, which was a poor prognostic factor (*p* = 0.026). All cases of IOFB and penetrating injuries were related to the LP group and had a better prognosis than rupture injury.

All 12 of the IOFB cases occurred in the workplace, with 11 cases due to metal origin and 1 case due to concrete. IOFB removal was successful in all cases. A study by Madhusdhan et al. reported that the cases were predominantly penetrating injuries and that the mechanism of injury was not significantly associated with the final visual acuity [11]. Our study confirmed that rupture was a mechanism of injury with a poor prognosis for the visual outcome (*p* = 0.026), which is in agreement with other studies that showed rupture was associated with a lower rate of both visual function and functional success rates than laceration [12, 23]. Additionally, these studies confirmed that both IOFB (other than metal pellets fired from a BB gun) and penetrating injuries resulted in better visual results and prognoses [12, 23]. Perforating injuries from explosions and gunshots were not observed in our current cases, as these are rare in the Japanese community.

In our study, patients with wounds that were smaller than 5 mm had a good prognosis for the visual outcome compared to patients with wounds that were larger than 5 mm (*p* = 0.0078), which is in accordance with several previous reports [14, 17, 18, 24, 25]. A larger wound reflects more extensive ocular tissue damage and a higher likelihood of posterior involvement. Additionally, Rofail et al. demonstrated that a laceration larger than 10 mm had a 14.49-fold risk of attaining a final VA of CF or worse compared with lacerations that were 1 to 5 mm [14].

Han and Yu established that a larger wound (> 10 mm) was related to a poorer final visual acuity [12]. These findings suggested that the size of the laceration had both therapeutic and prognostic implications, with an increase in the laceration length significantly correlated to a worse visual outcome (*p* < 0.001).

Our results also demonstrated that the zone of the injury was associated with the visual outcome. Wounds involving zone III had significantly poorer visual outcomes versus those involving zones I or II. This result is supported by previous studies, which reported a significant association between the posterior extension of the wound and a worse final VA [3, 11, 12, 18, 26–28]. Madhusudhan et al. reported that subjects with a wound extending posterior to the equator had 20 times the risk of having a final visual acuity less than 3/60 as compared to patients whose wounds were anterior to the recti insertions or restricted to the cornea [11].

Our study showed that a poor VA at the first visit was an important prognostic factor (*p* = 0.00001). A good initial VA was the strongest prognostic factor of a favorable final VA in both the univariate and multivariate analyses, similar to that reported by other numerous studies [4, 11–18, 23–25].

Han and Yu performed a multivariate analysis and demonstrated that an initial VA of LP or better resulted in an 18.2-fold chance of attaining visual survival, while CF or better resulted in a 2.81-fold chance of achieving functional success. This result suggests that a better initial VA reflects milder ocular tissue damage, which ensures a better visual outcome. In contrast, an initial VA of NLP suggests serious ocular tissue destruction, particularly of the retina and optic nerve [12].

Many previous studies have reported finding a significant correlation between the lenticular involvement and the visual outcome [12, 15, 23, 24, 29]. In our study, crystalline lens expulsion was observed in 11 eyes (5 in the LP group and 6 in the NLP group) and this group had a significantly poor prognosis for the visual outcome (*p* = 0.0003). In contrast, phakia, aphakia, and pseudophakia were not significant predictive factors for the visual outcome. Thus, expulsion of the crystalline lens can be used to document the degree or extensiveness of the injury and determine if it will affect other important structures in the eye, thereby leading to a worse visual outcome.

Our study found a history of PK in 4 (30.8%) of the 13 eyes in the NLP group and in 3 (6.5%) of the cases in the LP group. To the best of our knowledge, this is the first study to clarify that PK is a poor prognostic factor for open-globe injuries, and that there is a statistically significant association with the visual outcome (*p* = 0.017).

As previously discussed, we evaluated 12 IOFB cases that all occurred in the workplace. In all cases, the IOFB removal was successful, with 11 cases due to a metal origin and 1 cased due to concrete. All of these IOFB cases belonged to the LP group, and they did not show that the group had a poor predictive effect on the visual outcome. This may be due to the location of the IOFB, as only 1 case was present at the posterior pole nasal to the optic disc, while all of the others were distributed in the anterior chamber (3 cases), vitreous (2 cases), and peripheral retina (6 cases). Although we did not find the IOFB to be a significant predictive factor, its prevalence as the second major mechanism of injury suggest that it should carefully evaluated, especially if it is a preventable cause. Though there are mandatory regulations that have been designed to reduce the incidence of eye trauma, such as the use of protective glasses in the workplace, many people simply ignore these precautions. Thus, there needs to be a widespread increased public awareness about eye injuries, IOFB complications, and measures that can be taken in order to prevent eye trauma, especially among those workers who are exposed to such dangers during their normal workdays.

Our current study showed that both RD and VH were significantly poor prognostic factors for visual outcomes. These findings are in agreement with previous studies that also reported RD to be a poor prognostic factor for visual outcome [3, 12, 15, 30, 31]. Additionally, other previous studies have found VH to be a poor predictive factor for visual outcome [15, 23, 24, 29–31]. However, there were no cases of endophthalmitis in our current study, which may be due to the early closure of the wound and prompt initiation of antibiotics that was performed in all of our cases.

One of the challenges in the treatment of open-globe injuries is identifying the optimal timing for the ultimate reconstruction, namely vitrectomy. While it is clear that suture-closure of the wound for open-globe injuries should be performed as soon as possible, it is less clear whether vitrectomy should be performed during the same surgical session (primary comprehensive reconstruction) or deferred until a later time (staged approach) [32].

In this study, we concluded that eyes that underwent PPV as the primary procedure were significantly more likely to achieve a final vision of LP or better versus those that did not undergo the procedure (*p* = 0.033). PPV was the primary operation used in 29 (49.2%) of the cases, with 26 (89.7%) exhibiting a good visual outcome (LP group) and 3 (10.4%) failing to achieve a gain in their vision (NLP group). This is in agreement with previous studies that have demonstrated the efficacy of vitreoretinal surgery for treating open-globe injuries [12, 33–35]. In a retrospective, matched cohort study, De Juan et al. found no benefit of vitrectomy in the management of penetrating ocular injury [29]. It should be noted, however, that this is a relatively old study that was performed in 1983, which was well before the marked improvement of the PPV techniques and instrumentation that are currently available.

Another important feature in our current versus the previous studies is that we evaluated the number of open-globe injuries over a period of more than 5 years. The number of these injuries was less than one patient per month (59 cases/66 months), which indicates an awareness by the Japanese population with regard to the use of protective measures for preventing open-globe injuries.

The limitation of our study is related to insufficient documentation, especially with regard to the presence or absence of a relative afferent papillary defect (RAPD).

In conclusion, improvements in our knowledge of the pathophysiology of eye trauma and its prognostic factors, as well as advances in diagnostic and therapeutic techniques, have greatly improved the success rates for managing open-globe injuries. A better understanding of these prognostic factors may help provide our patients with better and more realistic expectations of their final VA. This study demonstrated that a poor VA at the first visit, a ruptured globe, zone III injuries, PK, RD, VH, and expulsion of the crystalline lens are considered to be poor prognostic factors for open-globe injuries. Patients with a wound smaller than 5 mm had a significantly better VA than the other groups with wounds larger than 5 mm. When patients with open-globe injuries had posterior segment involvement, PPV proved to be a good prognostic factor.
